# The Work of the Digestive Glands

**Published:** 1903-06

**Authors:** 


### Reviews of Books.

**The Work of the Digestive Glands.** Lectures by Professor J. P. PAWLOW. Translated by W. H. THOMPSON, M.D. Pp. xii. 196. London: Charles Griffin & Company. 1902.

This book contains a quantity of new and original work on the digestive functions; the conclusions have been arrived at by very ingenious and elaborate methods of experiment. One of the most interesting and novel points brought out by these observations is the very striking range of variability and adaptation to the varying needs of digestion displayed by the gastric and pancreatic secretions. Thus, although the acidity of the former appears to be constant when secreted, the total acidity of the gastric contents can vary by means of alterations in the quantity of gastric juice poured out. Further, if one kind of foodstuff predominates in, or forms exclusively, the diet of an animal for some time, the ferment concerned in the digestion of that foodstuff is increased in quantity, while the others diminish.

Possibly this is the explanation of the transitory dyspepsia which is apt to intervene on a sudden change from a long-accustomed diet.

The secretion of one digestive juice seems to stimulate by a beautiful mechanism the secretion of the next one, which, in a succeeding portion of the alimentary tract, is to carry the food a stage further in the process of digestion. Thus the water of the saliva stimulates a flow of gastric juice, and the secretion of H Cl by the stomach is a powerful stimulus to pancreatic secretion. The author suggests that the Na derived from the Na Cl broken up to form H Cl in the stomach, may be utilised in the production, shortly afterwards, of the alkaline pancreatic juice. The author looks upon the mechanism of secretion in stomach and pancreas as a reflex one, through stimulation of the peripheral nerves, and gives experiments to prove that the vagus is the nerve of secretion for both organs. The recent observations of Starling on the formation of *secretin* in the duodenum and its influence upon pancreatic secretion prove, however, that in this particular case the secretion is not a reflex nervous one, and it is possible that this may yet be proved for other secretions.

Of interest to medical practitioners are the importance of appetite, that is, of the "psychical factor," in promoting an adequate flow of gastric juice, and the justification of certain time-honoured procedures in matters of diet. We already owe to Sir Wm. Roberts's writings the recognition that many dietetic customs were based on sound physiological fact, and we learn again in this work that the medicinal use of bitters is justified by the flow of gastric juice they promote, that meat extracts form one of the most potent stimulants to this secretion, hence the *rationale* of beginning dinner with soup, and that the ending of the meal with sweats is useful, as, not being digested in the stomach, they make no further call upon this organ for digestion, whilst their agreeable taste promotes a fresh flow of gastric juice at a time when it would otherwise be falling off.

We have mentioned a few points of interest, but the book itself should be read by all medical practitioners: it is well written, most interesting and suggestive, and its perusal will lead to a better understanding of digestion, and to better treatment of its pathological disorders.

